# Risk factors for early seizure after revascularization in patients with moyamoya disease

**DOI:** 10.1186/s41016-022-00305-0

**Published:** 2022-12-27

**Authors:** Hongchuan Niu, Cunxin Tan, Kehan Jin, Ran Duan, Guangchao Shi, Rong Wang

**Affiliations:** 1 Department of Neurosurgery, Peking University International Hospital, Beijing, China; 2 Department of Neurology, Peking University International Hospital, Beijing, China; 3 Present address: Department of Neurosurgery, Beijing Tiantan Hospital, Capital Medical University, No.119 South 4th Ring West Road, Fengtai District, Beijing, 100070 China

**Keywords:** Moyamoya disease, Revascularization, Seizure

## Abstract

**Background:**

To investigate the risk factors for early seizure after revascularization in patients with moyamoya disease (MMD).

**Methods:**

A total of 298 patients with MMD diagnosed in our hospital from 2015 to 2018 were analyzed retrospectively. We summarized the characteristics of seizure after revascularization in patients with MMD and analyzed the predictors of early postoperative seizure.

**Results:**

We identified 15 patients with MMD who developed seizures within 1 week after revascularization. According to logistic regression analysis, age (OR: 1.04, 95% CI 0.998–1.086; *P* = 0.060) and infarct side (OR: 1.92, 95% CI 0.856–4.290; *P* = 0.113) were not significantly associated with incident early seizure. Postoperative infarction (OR: 12.89, 95% CI 4.198–39.525; *P* = 0.000) and preoperative cerebral infarction (OR: 4.08, 95% CI 1.267–13.119; *P* = 0.018) were confirmed as risk factors for early seizure.

**Conclusions:**

We believe that a history of preoperative infarction and new infarction are independent risk factors of early seizure in patients with MMD after revascularization.

## Background

Moyamoya disease is a type for cerebrovascular disease with unknown etiology, which is characterized by chronic progressive stenosis or occlusion of the ends of bilateral internal carotid arteries, anterior cerebral arteries, and the beginning of middle cerebral arteries, and is secondary to the formation of the abnormal vascular network in the skull base. It was first reported by Japanese scholars Suzuki J and Takaku in 1969 [1]. Both direct and indirect revascularizations or the combination of these two types results in a seizure. There are only a few studies on seizures after an MMD operation [2–7]. In the present study, we summarize the characteristics of early postoperative seizure in patients with MMD to estimate its risk factors.

## Methods

### Patient selection

We retrospectively analyzed all patients diagnosed with MMD (I67.5, ICD-10-CM code) in our hospital from 2015 to 2018. The inclusion criteria were as follows: (1) patients confirmed to have MMD as diagnosed by digital subtraction angiography (DSA) or magnetic resonance angiography (MRA) according to the guidelines of the MMD (Willis circle spontaneous occlusion) Research Committee in 2012 and (2) patients with seizure and abnormal EEG (epileptiform discharge of different degrees) after vascular reconstruction. Cases with moyamoya syndrome with autoimmune disease, Down syndrome, hyperthyroidism, neurofibromatosis, vasculitis, or other associated diseases were excluded from our study. The study was approved by the research ethics committee of Peking University International Hospital. All patients and their families gave informed consent before participating in the study.

### Imaging evaluation

All patients were scanned by computed tomography (CT) or magnetic resonance imaging (MRI) at admission and all patients underwent DSA after admission. According to the angiographic staging of MMD described by Suzuki and Takaku, MMD was classified on the basis of DSA. We used perfusion CT to assess cerebral blood flow (CBF). All the imaging data were independently reviewed by 2 neurologists who were blinded to the patient’s information. Any discrepancies were resolved by consensus or by a third party.

### Treatment strategy

The indications of MMD include (1) obvious cerebral ischemia or hemorrhage and (2) recurrent clinical symptoms caused by perfusion injury, including CBF reduction and insufficient reserve. The common operation methods are direct bypass and indirect bypass. Indirect revascularization includes brain dura temporal artery application (EDAs) and burr holes. Direct revascularization included end-to-side anastomosis of the branches of the superficial temporal artery (STA) and the cortical branches of the middle cerebral artery. Combined revascularization (indirect revascularization and direct revascularization on the same side) was analyzed as direct revascularization. For direct bypass, we chose the anterior branch of STA as the donor and the M4 branch of the middle cerebral artery as the recipient artery. After craniotomy of a small bone flap (3–5 cm in diameter) for the lateral fissure, we performed end-to-side anastomosis of donor and recipient arteries. Indocyanine green angiography confirmed the patency of the grafts. For encephaloduroarteriosynangiosis (EDAS), STA was exposed and then sutured to the brain surface. For multiple burr holes, 5–15 holes were placed in the hypoperfusion brain area, and the dura was simultaneously cut and separated at the same time. In our institute, we mainly perform direct vascular reconstruction in all patients, unless the donor or recipient vessels are too small to be anastomosed. In one operation, we performed only unilateral revascularization. For the choice of operative side, the symptomatic side or hypoperfusion side is the priority side of revascularization. All patients received antiepileptic drugs after revascularization.

### Evaluation of clinical results

According to the definition of an early traumatic seizure [8] and the international league against seizure guidelines, an early seizure was defined as a seizure that occurred within 7 days [9]. We collected early epileptic seizures that occurred within 1 week after the operation. An electroencephalogram (EEG) confirmed that the symptoms of seizures were recorded. According to the classification of epileptic seizures, the duration and types of seizures were recorded for analysis.

### Statistical analysis

Descriptive summaries were reported as mean ± standard deviation of consecutive variables and the frequency (percentage) of categorical variables. We used logistic regression to calculate the odds ratios (ORs) of 95% confidence intervals (CIs) and *P* value. All the data were statistically analyzed by correlation analysis, *χ*^2^ test, one-way ANOVA, and logistic regression to determine the risk factors that were related to the outcome of adverse seizure. Statistical software was used for the analysis of data.

## Results

**Fig. 1:**
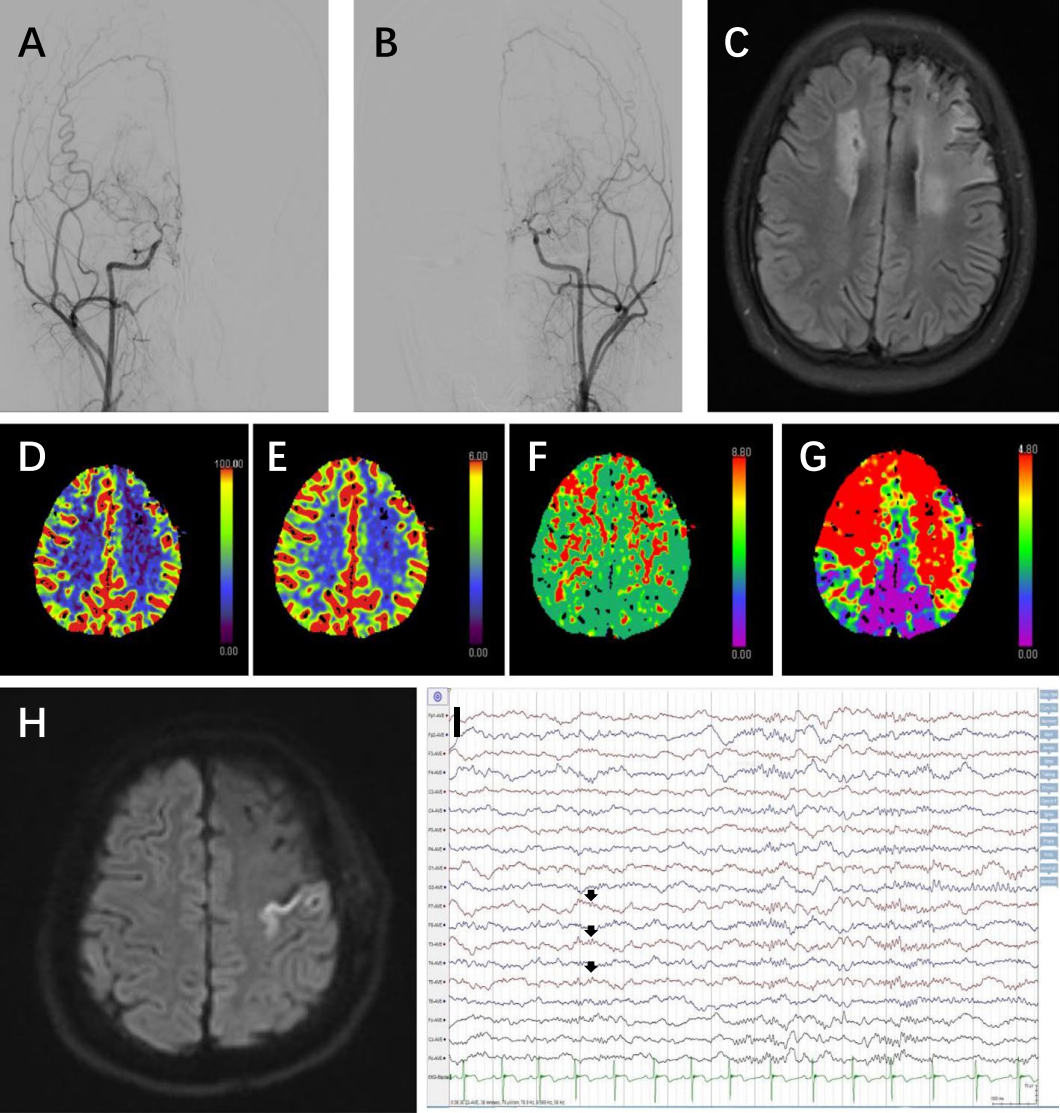
Case 9. Female, 14 years, left EDAS. One day after the operation, her right limb clonus and she was conscious. She was found to have cerebral infarction after the operation. **A**, **B** DSA showed occlusion of bilateral internal carotid arteries and formation of smoke-like vessels before operation. **C** Preoperative MRI revealed chronic cerebral infarction lesions in bilateral centrum semiovale and left frontal cortex. **D**–**G** Preoperative CTP (**D** CBF is roughly the same on both sides; **E** CBV is roughly the same on both sides; **F** MTT bilateral frontal lobe slows down; **G** Tmax bilateral frontal lobe slows down). **H** One day after operation, a high signal intensity of the left frontal lobe can be seen on the DWI sequence, and acute cerebral infarction is considered. **I** Postoperative VEEG showed that F7, T3, and T5 (left posterior temporal and frontal lobe) were visible Ɵ wave low amplitude slow wave activity (arrow)

From 2015 to 2018, 15 (5.0%) of 298 patients with MMD who underwent revascularization in Peking University International Hospital developed seizures after revascularization (Table 1). The summary of clinical characteristics of patients with or without seizures is as follows (Table 2): There were 6 males (40%) and 9 females (60%) who developed seizures after surgery. The sex ratio of epileptic patients and nonepileptic patients was similar (*P* = 0.711). The data of age were tested for normality. The average age of patients with early postoperative seizure was 43.00 ± 15.21 years, while the average age of the nonseizure group was 35.24 ± 15.14 years. There was no significant age difference between the two groups (*P* = 0.054). There is a whole record of one typical patient as shown in Fig. 1.

**Table 1:** Clinical data of early epilepsy after revascularization in MMD patients

No.	Gender	Age	Cerebral infarction before op	Revascularization	Cerebral infarction after op	Seizure onset after op (d)	Seizure types after op
1	M	41	Y	Bypass+EDAS	Y	1	SPS
2	F	51	Y	Bypass+EDAS	N	5	GTCS
3	F	32	Y	Burr holes	N	1	CPS
4	F	54	Y	EDAS	Y	1	CPS
5	M	50	Y	Bypass+EDAS	N	3	GTCS
6	F	32	N	Bypass+EDAS	N	5	GTCS
7	M	53	Y	Bypass+EDAS	N	1	SPS
8	F	45	Y	EDAS	Y	1	SPS
9	F	14	Y	EDAS	Y	1	SPS
10	M	53	Y	Bypass+EDAS	Y	1	SPS
11	M	57	Y	Bypass+EDAS	N	6	SPS
12	F	45	N	EDAS	Y	6	SPS
13	F	55	N	Bypass+EDAS	Y	1	CPS
14	F	55	Y	Bypass+EDAS	N	1	SPS
15	M	8	N	EDAS	N	6	SPS

*M* Male, *F* Female, *Y* Yes, *N* No, op Operation, *EDAS* Encephalo-duro-arterio-synangiosis, *SPS* Simple partial seizures, *CPS* Complex partial seizures, *GTCS* Generalized tonic–clonic seizures

**Table 2:** Clinical characteristics of patients with EP and without EP after the operation

	Patients with EP after the operation	Patients without EP after the operation	*P*
Number of patients	15	283	
Age (years)	43.00±15.208	35.24±15.140	0.054
Male	6	127	0.711
Infraction before op	10	102	0.011
Infraction side before op			0.042
Left	4	51	
Right	1	33	
Both	5	18	
Hemorrhage before op	0	68	0.031
EP before op	3	23	0.806
History of EP (months)	4.52±36.814	3.20±12.394	0.89
SE before op	1	4	0.025
Surgical modalities			0.907
Bypass	0	1	
EDAS	5	77	
Bypass+EDAS	9	187	
Burr holes	1	8	
EDAS+burr holes	0	9	
Bypass+EDAS+burr holes	0	1	
Postoperative hyperperfusion syndrome	0	5	0.604
Infraction after op	7	18	0

*EP* Epilepsy, *op* Operation, *SE* Status epilepticus, *EDAS* Encephalo-duro-arterio-synangiosis

There were 112 patients with cerebral infarction before the operation; 10 of them had epileptic seizures after the operation, and the results were significantly different (*P* = 0.011). Four of the 10 patients with cerebral infarction were left, one was right, and five were bilateral. According to the *χ*^2^ test, bilateral cerebral infarction was observed before the operation, and the incidence of early epileptic seizures was high (*P* = 0.042). Among 68 patients with MMD, none of them had an early seizure after the operation. According to the correlation analysis, a negative correlation was observed between preoperative cerebral hemorrhage and postoperative early seizure (*P* = 0.031). There were 26 patients with previous epileptic history before the operation, but only 3 patients had epileptic seizures after the operation, and there was no significant difference (*P* = 0.806). The average medical history of 3 patients was 4.52 ± 36.814 months, the average medical history of 23 patients with early postoperative seizures was 3.20 ± 12.394 months, and the difference was not statistically significant (*P* = 0.890). Five patients had status epileptecus before the operation, and one of these patients had a seizure postoperatively (*P* = 0.025). In the seizure group, 9 patients (60%) underwent direct reconstruction, 5 patients (33.3%) underwent EDAs, and 1 patient (6.7%) underwent burr holes. There was no significant difference in the choice of surgical modifications. After the operation, 5 patients had hyperperfusion syndrome, and none of them had early seizures. After calculation, the theoretical frequency of postoperative hyperperfusion and postoperative hemorrhage was less than 1. Therefore, we conducted Fisher’s exact test for these two factors in the next step and found that the hyperperfusion *P*=1.000 and the postoperative hemorrhage *P*=1.000 both of which had no statistical difference. There were 25 patients with new cerebral infarction after the operation, and 7 (28.0%) of them had early seizures. The statistical analysis revealed a positive correlation between new cerebral infarction and early seizure (*P* = 0.000). We followed up 15 patients with early epilepsy and found that 13 patients with seizures could be effectively controlled after conservative treatment with antiepileptic drugs. One patient still had a simple partial seizure after treatment, but the frequency of the seizure was very low. Another patient suffered from early epilepsy and severe cerebral infarction, leading to death.

The analysis of risk factors of early seizure postoperatively is shown in Table 3. Logistic regression revealed that age (OR: 1.04, 95% CI 0.998–1.086; *P* = 0.060) and infarct side (OR: 1.91, 95% CI 0.856–4.290; *P* = 0.113) were not significantly associated with incident early seizure. Postoperative infarction (OR: 12.88, 95% CI 4.198–39.525; *P* = 0.000) and preoperative cerebral infarction (OR: 4.07, 95% CI 1.267–13.119; *P* = 0.018) were identified as risk factors for early seizure.

**Table 3:** Variables of factors associated with early epilepsy after revascularization analyzed by logistic regression

Variable	*B*	S.E.	Wald	*v*	*P*	Exp(*B*)	95% CI	95% CI
							Lower limit	Upper limit
Constant	−4.527	0.955	22.481	1	0.000	0.011		
Age	0.040	0.021	3.531	1	0.060	1.041	0.998	1.086
Prior infarction side	0.651	0.411	2.505	1	0.113	1.917	0.856	4.290
Postoperative infarction	2.556	0.572	19.965	1	0.000	12.882	4.198	39.525
Prior infarction	1.405	0.596	5.554	1	0.018	4.077	1.267	13.119
EP	−0.260	1.057	0.061	1	0.805	0.771	0.097	6.117

*EP* Epilepsy, *B* Beta, *CI* Confidence interval

## Discussion

In this study, the average ratio of male to female was 1:1.22 (male 134:female 164), with 57 children and 241 adults, and the incidence of preoperative seizure was approximately 8.72%. This is basically consistent with the results of previous studies. For the 941 cases of MMD in Japan, the female to male ratio was 1.98:1. The two peaks of age of onset were 5–9 years and about 40 years. The incidence of seizures was 4% [10]. For children with MMD, Ma reported in 2017 that the proportion of men and women with seizures was approximately 1:0.87, and there was no difference in the sex ratio, with an incidence of 18.1% [11].

Patients with MMD report transient ischemic attack (TIA) before the operation, but it is difficult to distinguish from some epileptic attacks in the form of expression. It was found that the occurrence of cerebral infarction before the operation and new infarction after the operation were the risk factors of early seizure in patients with MMD after revascularization. There are two mechanisms that may lead to early and late epileptic seizures after neurosurgery. One mechanism is the extravasation of blood vessels. Iron released from hemoglobin reacts with hydrogen peroxide in the surrounding tissues to produce free radicals, which may lead to early epileptic seizures and the formation of epileptic foci. Hydrogen peroxide reduces the inhibition of γ-aminobutyric acid (GABA)-mediated cortical and thalamic neurons. Free radicals can promote the excitability of thalamic cortical circuits by changing the neurotransmitter mediated by β-aminobutyric acid. Repeated exposure to free radicals will lead to the formation of epileptic foci. Another mechanism may be through a disorder of ion balance in the cell membrane caused by ischemia or hypoxia. The decrease of high energy reserve, such as adenosine triphosphate under the condition of ischemia or hypoxia, further leads to the disorder of ion balance on the cell membrane; this is because the ratio of intracellular and extracellular K+ concentration decreases, whereas the ratio of sodium ion concentration increases. This imbalance reduces the hyperpolarization of neurons that may cause transient or early seizures. The imbalance of sodium ions is mainly mediated by the outflow of Ca2+-independent glutamate during ischemia [12].

Choi et al. found that 4/7 of the patients with MMD had a seizure with hypoperfusion [13], and stroke and hypoperfusion would lead to cerebrovascular reactive disorder, followed by hypoxia of brain tissue and seizure. Blood flow reconstruction can improve the perfusion of brain tissue and prevent seizures [13]. According to Mikami et al., a moyamoya seizure is a typical type of seizure after stroke. However, in a previous study, the severity of vascular stroke shown by MRA was not related to seizure [14]. The prognosis of MMD epileptics with abnormal CT findings was poor [15]. Ma et al. suggested that preoperative cerebral infarction could not predict the recurrence of seizure [11].

Ma et al. found that the duration of epileptic attack before the operation was an independent risk factor for seizure recurrence after revascularization of children with MMD [11], and revascularization should be performed immediately after the diagnosis of seizure. In our study, there was no correlation between preoperative seizure history and postoperative seizures. In this group of patients, we found that the preoperative status of seizures in patients with MMD may be related to the early postoperative seizures as shown by one-way ANOVA. A few other studies have reported on a seizure caused by MMD, but in other studies, it was related to cerebrovascular diseases. According to Englot et al., the seizure time was less than 1 year, which is a predictor of seizure in a patient with cavernous malformation [16]. According to Falero and León, the short seizure time of arteriovenous malformation (AVM) was statistically significant with class I after the operation [17]. Liu et al. believe that the long duration of AVM indicates poor prognosis [18].

In this group of cases, the seizure forms of patients with seizures before the operation include simple partial seizures (SPS), complex partial seizures (CPS), and generalized tonic–clonic seizures (GTCS). Among the 23 epileptic patients before the operation, 12 had SPS attack and 6 had CPS attack. Some of them had high incidence, which may be related to focal infarction or ischemic focus before operation. However, we found that patients with a persistent state of seizure before the operation are more likely to have an early seizure after undergoing revascularization. Few researchers examined the relationship between seizure form and seizure after operation for MMD, while in other cerebrovascular disease studies, the seizure form is related to the prognosis of patients; moreover, there are also contrary findings. GTCS attack in cavernous malformation (CM) patients indicates poor prognosis [19–22]. According to Falero and León, GTCS attack in AVM indicates a good prognosis [17]. In patients with MMD, there was no correlation between attack pattern and prognosis.

In the present study, we did not find that the choice of operation mode was related to the early postoperative seizure. For patients with ischemic MMD and a high risk of preoperative infarction, we chose EDAS operation with a shorter operation time to reduce the risk of anesthesia and operation. If there are receptor vessels with appropriate thickness and flow, the bypass operation is more feasible. Therefore, there is a certain bias in the selection of the operation method, which is often compared with decision-making. According to Choi et al., EDAS is safe and effective to prevent postoperative seizure [13]. Ma et al. in 2017 found no difference between the two surgical methods (direct/indirect) in the prevention of postoperative seizure [11]. There are still many concerns associated with the specific operation. In this group, the blood supply reconstruction operation mode involves cutting the skin craniotomy and milling the bone flap along the superficial temporal artery. Some units use the frontotemporal craniotomy and mill the bone flap. However, it is unclear (1) whether there is any influence of the size of the bone window exposing the brain tissue area on the postoperative seizure, (2) whether there is stimulation of the connective tissue around the superficial temporal artery, (3) whether there is traction and contusion of brain tissue during the operation, and (4) whether there is subarachnoid hemorrhage during the operation. This information may have an impact on the postoperative seizure, but no detailed data can be used for in-depth statistics.

Hyperperfusion of brain tissue after STA–MCA bypass can cause transient neurological dysfunction, including postoperative seizure [23]. However, there are similar biological mechanisms between postoperative seizure and postoperative hyperperfusion syndrome [12, 24]. Postoperative high perfusion syndrome can cause headache, eye and facial pain, epileptic seizure, and focal neurological dysfunction secondary to brain edema. The pathological mechanism may be the impairment of cerebrovascular self-regulation function, which makes it difficult to regulate the blood flow from the superficial temporal artery to the middle cerebral artery after bypass surgery. In addition, oxygen free radicals produced during reperfusion after revascularization may damage cerebral vessels, leading to the occurrence or aggravation of high perfusion. The intrinsic response of some revascularization, such as the increase of vascular permeability, may be related to high perfusion after revascularization in MMD patients. Long-term cortical ischemia can induce the overexpression of angiogenic factors and extracellular matrix proteins, thus promoting the formation of new blood vessels and increasing the permeability of blood vessels [25].

According to the new National Institute for Health and Care Excellence guidelines, carbamazepine and lamotrigine are the first-choice drugs for focal seizure [26]. Most of the cases of moyamoya occur in young women. For the long-term control of moyamoya seizure, lamotrigine and levetiracetam can be used as the first-choice drugs in consideration of tolerance and family burden [14].

The present study has several limitations: first, although the total number of patients with MMD included in this study is quite large, the number of seizure cases after the operation is insufficient. To confirm our conclusion, a larger sample size and randomized design of future research are needed. Second, it is impossible to collect more detailed information about the operation details and techniques. Perhaps more detailed observation indicators should be taken in the future research design, in order to obtain more detailed conclusions.

## Conclusions

Our study showed that the history of preoperative infarction and new postoperative infarction are independent risk factors for early postoperative seizure in patients with MMD.

## Data Availability

All data generated or analyzed during this study are included in these published articles.

## Abbreviations

MMD: Moyamoya disease
ICA: Internal carotid artery
MCA: Middle cerebral artery
ACA: Anterior cerebral artery
TIA: Transient ischemic attack
EDAS: Encephaloduroarteriosynangiosis
EMS: Encephalomyosynangiosis
STA–MCA: Superficial temporal artery–middle cerebral artery
DSA: Digital subtraction angiography
